# Integrated Molecular Profiling of Human Gastric Cancer Identifies DDR2 as a Potential Regulator of Peritoneal Dissemination

**DOI:** 10.1038/srep22371

**Published:** 2016-03-03

**Authors:** Junji Kurashige, Takanori Hasegawa, Atsushi Niida, Keishi Sugimachi, Niantao Deng, Kosuke Mima, Ryutaro Uchi, Genta Sawada, Yusuke Takahashi, Hidetoshi Eguchi, Masashi Inomata, Seigo Kitano, Takeo Fukagawa, Mitsuru Sasako, Hiroki Sasaki, Shin Sasaki, Masaki Mori, Kazuyoshi Yanagihara, Hideo Baba, Satoru Miyano, Patrick Tan, Koshi Mimori

**Affiliations:** 1 Department of Surgery, Kyushu University Beppu Hospital, 4546 Tsurumihara, Beppu 874-0838, Japan; 2 Department of Gastroenterological Surgery, Graduate School of Medical Sciences, Kumamoto University, 1-1-1 Honjo, Kumamoto 860-8556, Japan; 3 Bioinformatics Center, Institute for Chemical Research, Kyoto University, Gokasyo, Uji, Kyoto 611-0011, Japan; 4 Human Genome Center, Institute of Medical Science, University of Tokyo, 4-6-1 Shirokanedai, Minato-ku, Tokyo 108-8639, Japan; 5 Cancer and Stem Cell Biology Program, Duke-NUS Graduate Medical School, College Road, Singapore 169857, Singapore; 6 Department of Gastroenterological Surgery, Osaka University Graduate School of Medicine, 2-2 Yamadaoka, Suita, Osaka 565-0871, Japan; 7 Department of Surgery I, Oita University Faculty of Medicine, 1-1 Idaigaoka, Yufu, Oita 879-5593, Japan; 8 Gastric Surgery Division, National Cancer Center Hospital, 5-1-1 Tsukiji, Chuo-ku, Tokyo 104-0045, Japan; 9 Department of Surgery, Hyogo College of Medicine, 1-1 Mukogawa, Nishinomiya 663-8501, Japan; 10 Department of Translational Oncology, National Cancer Center Research Institute, 5-1-1 Tsukiji, Chuo-ku, Tokyo 104-0045, Japan; 11 Omori Red Cross Hospital, 4-30-11 Chuo, Ohta-ku, Tokyo 143-8527, Japan; 12 Division of Translational Research, Exploratory Oncology Research & Clinical Trial Center, National Cancer Center, 6-5-1 Kashiwanoha, Kashiwa 277-8577, Japan

## Abstract

Peritoneal dissemination is the most frequent, incurable metastasis occurring in patients with advanced gastric cancer (GC). However, molecular mechanisms driving peritoneal dissemination still remain poorly understood. Here, we aimed to provide novel insights into the molecular mechanisms that drive the peritoneal dissemination of GC. We performed combined expression analysis with *in vivo-*selected metastatic cell lines and samples from 200 GC patients to identify driver genes of peritoneal dissemination. The driver-gene functions associated with GC dissemination were examined using a mouse xenograft model. We identified a peritoneal dissemination-associated expression signature, whose profile correlated with those of genes related to development, focal adhesion, and the extracellular matrix. Among the genes comprising the expression signature, we identified that discoidin-domain receptor 2 (*DDR2*) as a potential regulator of peritoneal dissemination. The *DDR2* was upregulated by the loss of DNA methylation and that *DDR2* knockdown reduced peritoneal metastasis in a xenograft model. Dasatinib, an inhibitor of the *DDR2* signaling pathway, effectively suppressed peritoneal dissemination. *DDR2* was identified as a driver gene for GC dissemination from the combined expression signature and can potentially serve as a novel therapeutic target for inhibiting GC peritoneal dissemination.

Gastric cancer (GC) is the fourth-most common human malignant disease and the second-leading cause of cancer-related deaths worldwide, with a particularly high incidence in East Asia^1^,^2^. The malignant potential of GC is characterized biologically by the dissemination of cancer cells from the primary site throughout the peritoneal cavity, in contrast to other tumor types that are spread hematogenously. Peritoneal dissemination is the most frequent cause of death in patients with advanced GC^3^. However, the mechanisms underlying peritoneal dissemination have not yet been specified, and advanced GC remains an incurable condition.

Previously, we established 4 cell lines with a high capability for peritoneal dissemination using 12 cycles of orthotropic transplantation directly into the gastric walls of nude mice and harvesting cells from ascites after peritoneal dissemination^4^,^5^. The phenotypes of these *in vivo-*selected GC cell lines resemble those in GC patients with peritoneal dissemination; thus, we were motivated to study the gene signatures of these cells to clarify the molecular mechanism of dissemination. Recent transcriptomic analyses of breast cancer, clear cell renal carcinoma, and colon cancer^6^,^7^,^8^,^9^,^10^ have successfully identified *in vivo* gene-expression signatures associated with a high risk of metastasis and poor overall survival. We applied computational bioinformatics analysis to determine the gene-expression signature related to GC peritoneal dissemination. To clarify the mechanisms of peritoneal dissemination in GC, we performed combined expression analyses of metastatic cell lines established from a peritoneal dissemination-xenograft mouse model and a clinical dataset of 200 GC patients.

## Results

### Establishment of a gene signature associated with peritoneal dissemination by comparison between resected primary tumors and *in vivo*-established cell lines in GC

The human parental cell lines HSC-44PE, HSC-39, and HSC-58 were derived from patients with gastric scirrhous cancer. The cell lines 44As3Luc, 39As8Luc, 58As1Luc, and 58As9Luc showing high metastatic potential were derived from the parental cell lines following 12 cycles of direct orthotropic transplantation to the gastric walls of nude mice and gathering cells from the ascites (Supplementary Fig. 1a–c)^11^.

**Figure 1:**
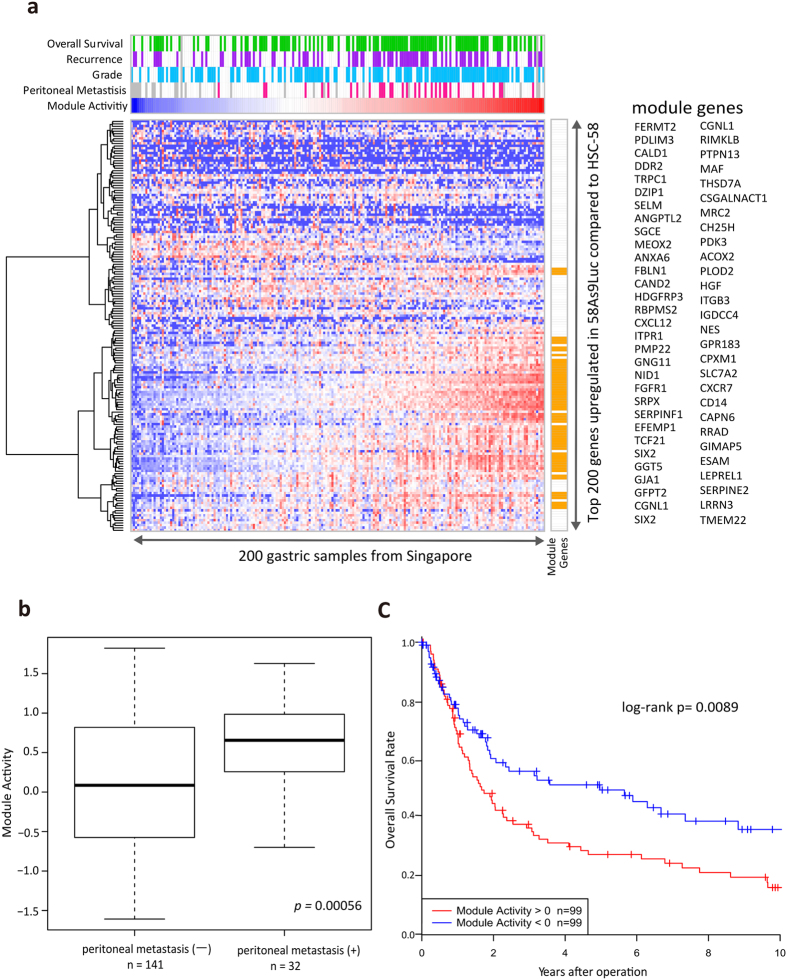
Establishment of the peritoneal dissemination signature in gastric cancer. (**a**) EEM analysis was used to compare cell lines and clinical samples, which showed that a gene expression signature from the 58As9Luc cell line harbored a coherently expressed subset within the clinical gene set. Hierarchical clustering of primary tumor tissue from a cohort of 200 gastric cancer patients was performed using the top 200 gene sets (i.e. with highest expression) in 58As9 cells, compared with the parental HSC-58 cell line. Assuming that the 57 signature genes (right yellow box and genes list) in the coherent subset were clinically relevant, we extracted them as the gastric dissemination expression signature (GDES). The clinical characteristics of gastric cancer patients are shown at the top of the figure. Overall survival: patients that died following surgery are represented in the green box. Recurrence: patients showing recurrence are represented in the purple box. Grade: patients with a diffuse-type grade according to the Lauren classification are represented in the blue box. Peritoneal metastasis: patients with peritoneal tumors or who were cytology-positive at the time of surgery are represented in the red box. Data deficiencies are represented in the gray box. (**b**) The high module activity of the GDES significantly associated with peritoneal metastasis. (**c**) Patients with high GDES module activity showed significantly poorer overall survival rates, compared to those with low GDES activity.

To identify genes that drive peritoneal dissemination, we performed expression analysis and obtained gene sets that were differentially activated in 4 metastatic cell lines, compared to their parent cell lines. To examine the clinical relevance of the *in vitro*-derived expression signatures, we subsequently performed extraction-of-expression modules (EEM) analysis, which tested the functionality of the input gene sets on the basis of their coherence^12^. Our EEM analysis combined with an expression dataset obtained with 200 resected GCs from a Singapore cohort^13^ indicated that the 58As9 cell line harbored a coherently expressed subset of significant size (Supplementary Fig. 2). Fifty-seven genes were analyzed using the EEM method, based on the expression profile observed in the 58As9 cell line. Considering the possibility that these 57 genes are clinically relevant, we extracted them as a gastric-dissemination expression signature (GDES, Fig. 1a). We found that patients harboring peritoneal metastases tended to show a more pronounced GDES (Fig. 1b), and patients with higher GDESs had a significantly poorer prognosis (Fig. 1c).

### Correlation of module genes with focal adhesion and extracellular matrix gene sets

**Figure 2:**
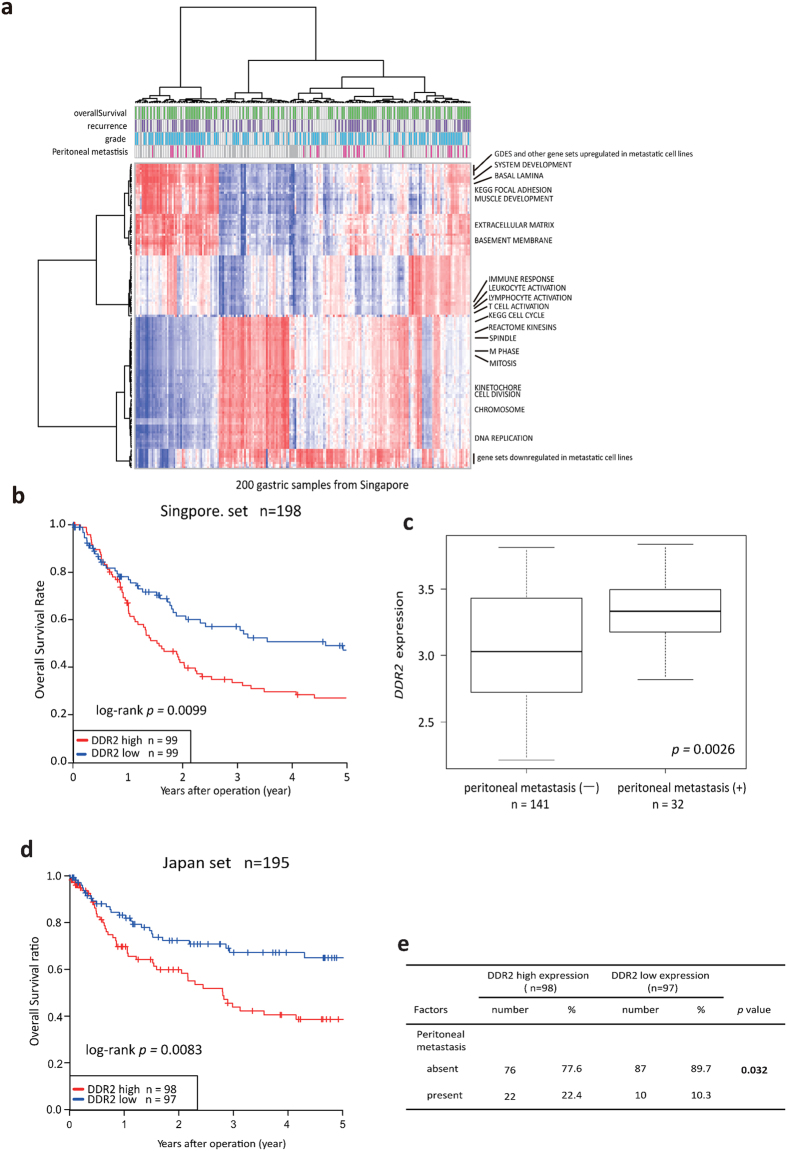
DDR2 expression was associated with peritoneal dissemination and poor prognosis in gastric cancer. (**a**) EEM-deduced activity profiles of GDES and other gene sets were evaluated by clustering analysis. (**b**) Kaplan–Meier survival curves for the 198 patients in the Singaporean cohort, with classification based on DDR2 mRNA expression levels measured using a microarray. (**c**) DDR2 expression was significantly higher in gastric cancer patients with peritoneal metastasis (Singaporean cohort). (**d**) Kaplan–Meier survival curves for the 195 patients in the Japanese cohort, as measured by qRT-PCR. (**e**) High DDR2 expression associated with gastric cancer patients that developed peritoneal metastasis, as determined by performing a Pearson’s chi-square test (Japanese cohort).

Next, we employed EEM analysis to study 1,981 gene sets that are registered with the Gene Ontology Consortium and 40 curated Cp entries in MSigDB (http://www.broadinstitute.org/gsea/msigdb/index.jsp), for a total of 2,021 gene sets. Among these gene sets, 139 were significantly associated with peritoneal dissemination using a threshold of p < 1.0e−6, and the module activities of these 139 gene sets were subjected to clustering analysis. By performing clustering analysis of the GDSE and miscellaneous gene sets, we found that the activities associated with the GDES positively correlated with the expression of development-related genes, focal-adhesion genes, which are fundamental for invasion and migration, and extracellular matrix (ECM)-related genes which are essential components of pre-metastatic and metastatic niches^14^,^15^ (Fig. 2a). In contrast, the activities associated with the GDES negatively correlated with the expression of cell cycle-related genes and genes downregulated in metastatic cell lines.

### DDR2 expression was epigenetically regulated and associated with peritoneal dissemination and poor prognosis in GC

**Figure 3:**
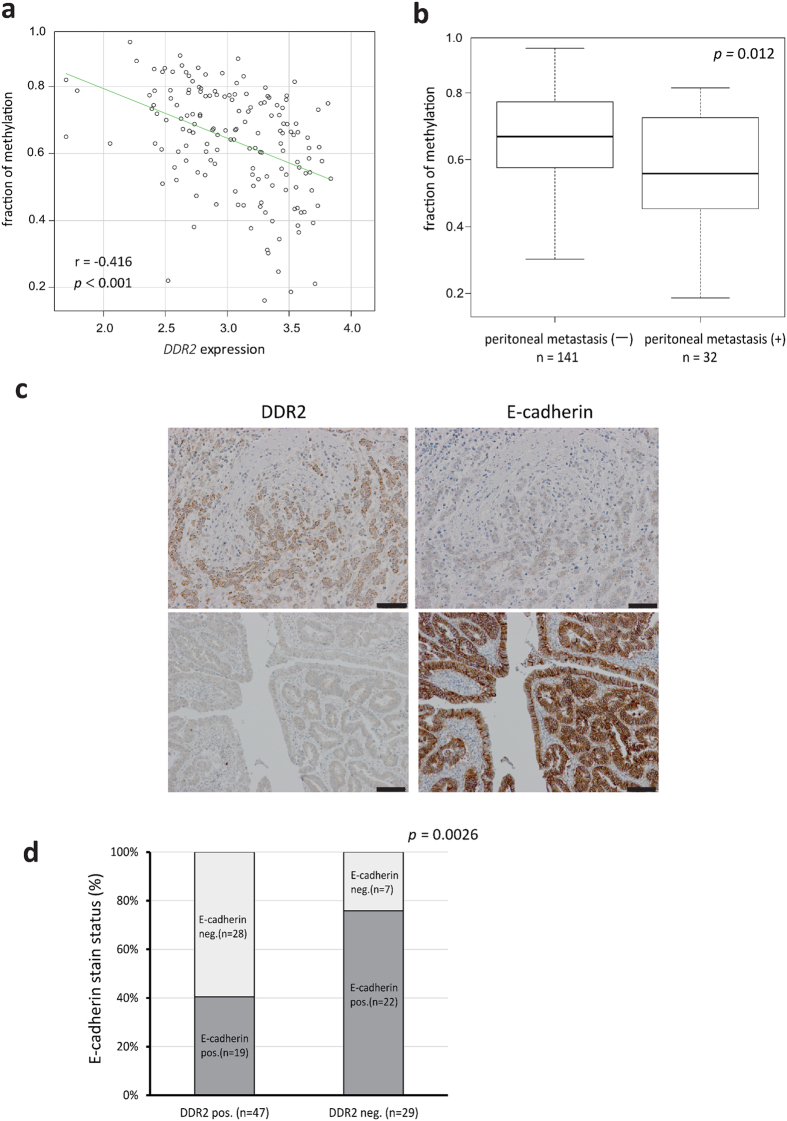
DDR2 overexpression was associated with DNA demethylation and epithelial-mesenchymal transition in gastric cancer. (**a**) Correlation plot between DDR2 expression and the fraction of methylation at the promoter region of DDR2 in 173 gastric cancer samples from the Singaporean cohort. (**b**) Box plot of the fraction of DDR2 promoter methylation between patients, with or without peritoneal metastasis. (**c**) Representative images of DDR2 and E-cadherin protein expression in gastric cancer cells. Scale bars, 100 μm. (**d)** DDR2 and E-cadherin expression were inversely correlated by immunohistochemical analysis of 76 gastric cancer samples.

The GDES contained several ECM-related genes. Among these genes, we focused on a type-I collagen receptor tyrosine kinase, discoidin domain receptor 2 (*DDR2*), which was one of the most highly expressed genes in 58As9 cells, compared with the parental cell line (31-fold higher). High *DDR2* expression significantly associated with poor prognosis and peritoneal dissemination in the GC samples from Singaporean and Japanese cohorts (Fig. 2b–e). We also found an inverse correlation between *DDR2* mRNA expression and DNA promoter methylation (Fig. 3a). Low promoter methylation was also associated with peritoneal dissemination (Fig. 3b). We performed immunohistochemistry studies of E-cadherin and DDR2 expression on surgically resected GC and found that DDR2 expression inversely correlated with that of E-cadherin (Fig. 3c,d). Taken together, these data suggested that epigenetically regulated DDR2 upregulation induced the epithelial–mesenchymal transition (EMT), which contributes to peritoneal dissemination in GC.

### Stable suppression of DDR2 reduced the cell proliferation rate, migration, and invasion and peritoneal dissemination

**Figure 4:**
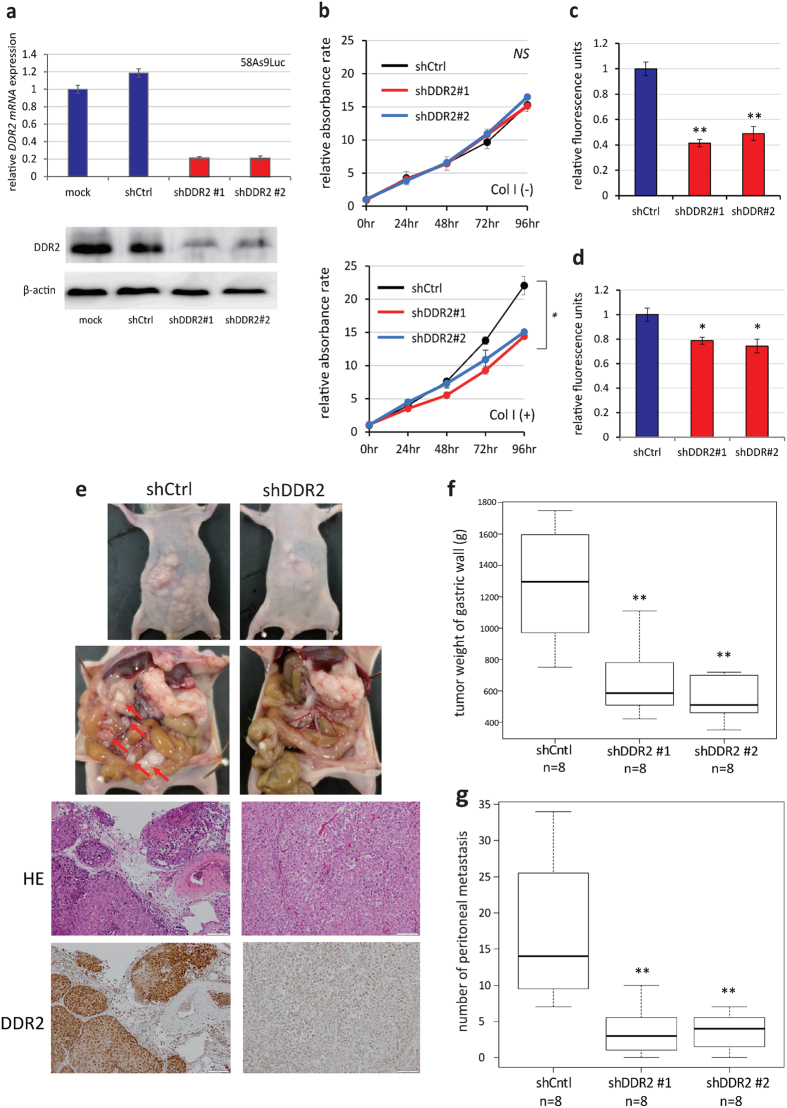
DDR2 inhibition reduced gastric cancer peritoneal metastasis *in vitro* and *in vivo*. (**a**) DDR2 expression was suppressed in 58As9Luc cells by 2 independent shRNAs. (**b**) DDR2 knockdown resulted in lower cell proliferation on collagen I-coated 96-well plates, but did not affect cell proliferation on normal plates. (**c**,**d**) Effects of DDR2 on (**c**) cancer cell migration and (**d**) invasion. (**e**) DDR2-knockdown 58As9Luc cells were orthotopically transplanted into the stomach walls of nude mice. (**f**,**g**) The normalized weight of gastric wall tumors (**f**) and the number of disseminated metastatic tumors (**g**) at 28 days post-transplantation with 58As9Luc cells with silenced DDR2 expression. Control, DDR2 sh#1, and sh#2: n = 8. *p < 0.05, **p < 0.01.

Next, we examined the involvement of DDR2 in peritoneal dissemination *in vitro*. We confirmed that DDR2 expression was elevated in 58As9 cells, compared with that in the parent cell line (HSC-58) by real-time quantitative reverse-transcription PCR (qRT-PCR), western blotting, and immunohistochemistry (Supplementary Fig. 3a–c). To examine the function of DDR2 in 58As9 cells, we performed knockdown experiments using 2 independent short hairpin RNAs (shRNAs), as shown in Fig. 4a. *DDR2* knockdown did not affect cell proliferation in the absence of collagen I, but significantly suppressed cell proliferation in the presence of collagen I (Fig. 4b). *DDR2* knockdown also attenuated the capacity of 58As9 cells for invasion and migration (Fig. 4c,d). We also performed knockdown experiments using the MKN7 GC cell line, which had high *DDR2* expression, and obtained similar results (Supplementary Fig. 4a–d).

Moreover, we performed an orthotopic transplantation of *DDR2-*knockdown 58As9 cells in the stomach walls of nude mice. At 28 days post-orthotopic transplantation, we found that *DDR2* knockdown reduced the number of disseminated metastases and tumor weights in the gastric walls (Fig. 4e–g). Based on these results, we tested whether *DDR2* could serve as a therapeutic target for GC with peritoneal dissemination.

### Dasatinib inhibited peritoneal dissemination via Src targeting in mice

**Figure 5:**
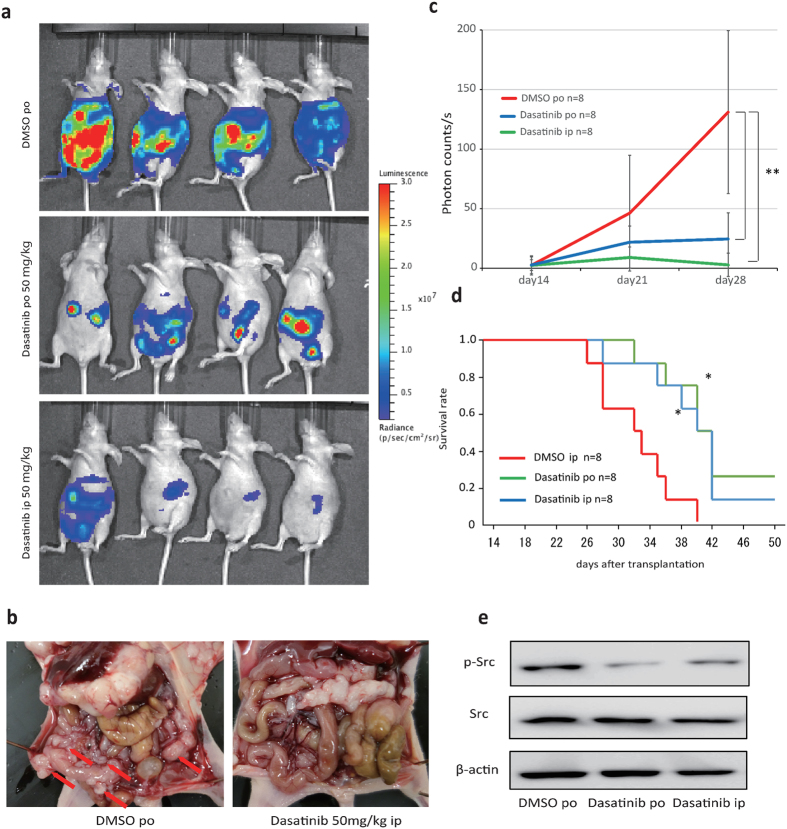
Dasatinib inhibited peritoneal dissemination. (**a**) Mice were treated orally and intraperitoneally with dasatinib at 50 mg/(kg·day) or a vehicle control, beginning at day 14 post-transplantation with 58As9Luc cells into the gastric wall. At day 28 post-transplantation, the mice were visualized using the IVIS system. (**b**) Macroscopic images showed an enlarged peritoneal cavity and metastatic nodules by controlMock3 and shDDR2. Arrowheads show nodules. (**c**) Normalized bioluminescence photon flux of disseminated peritoneal tumors at day 28 post-transplantation, as visualized by the IVIS system. The control and orally plus intraperitoneally administered dasatinib groups each contained n = 8 mice. **p < 0.01. (**d**) Survival curves showed that survival rate in dasatinib-treated mice was significantly higher (*p < 0.05) than that in control mice. (**e**) Src and Src phosphorylation, as measured by immunoblotting.

To assess the utility of DDR2 as a therapeutic target, we used dasatinib, an oral multi-BCR/Abl and Src family tyrosine kinase inhibitor that also inhibits DDR2^16^,^17^. The therapeutic effects of dasatinib on peritoneal dissemination were tested in a xenograft mouse model. We transfected the pGL4.51 (luc2/CMV/Neo) vector into 58As9 cells to evaluate the *in vivo* effects of dasatinib with an IVIS imaging system. Both orally and intraperitoneally administered dasatinib effectively suppressed the peritoneal metastasis of GC (Fig. 5a–c), leading to significantly longer survival times in dasatinib-treated mice than those in control mice (Fig. 5d). We examined the xenografts after treatment, which showed reduced Src-phosphorylation levels following both oral and intraperitoneal dasatinib administration (Fig. 5e).

## Discussion

By a combined analysis of *in vitro* and clinical datasets, we identified a GDES comprising genes whose expression levels are associated with gastric peritoneal dissemination. Among the genes in the GDES, our experiments showed that *DDR2* was responsible for peritoneal dissemination. Moreover, we found that dasatinib, a pre-existing drug that inhibits DDR2 kinase function, could suppress peritoneal dissemination.

In the clinical expression dataset, the GDES significantly correlated with the ECM signature. This observation suggested the importance of the tumor microenvironment niche in gastric peritoneal dissemination. Stromal collagen fibers perpendicularly aligned to the tumor boundary can foreshadow local invasion, intravasation, and metastasis^18^,^19^ , possibly by inducing the EMT and promoting a local stem-cell like phenotype^20^,^21^,^22^. The ECM is a complex scaffolding of proteins that provides architectural support for cell and tissue organization. In turn, cells can adhere to collagen or other ECM molecules via different types of receptors at distant sites^23^,^24^. The current comprehensive analysis strongly suggested that the ECM signature was responsible for peritoneal dissemination and, thus, could serve as a therapeutic target for advanced GC. Collagen can bind GC cells to promote proliferation, invasion, and migration^25^,^26^.

Based on the GDES, we focused on *DDR2* because it is a receptor tyrosine kinase activated by the binding of fibrillar collagen. Recently, it was reported that *DDR2* stabilized SNAIL1 to reduce E-cadherin protein levels and promoted the EMT in breast cancer cells^27^. Recent data revealed that *DDR2* mutations in lung squamous cell carcinoma could be therapeutically targeted by dasatinib^17^. In GC, *DDR2* mutations exist at a rate of 3.7% (8/219; The Cancer Genome Atlas data), which is similar to that observed in lung squamous cell carcinoma. Considering the data from this database and the current study results collectively, we expect that not only DDR2 overexpression, but *DDR2* mutations, could serve as promising therapeutic targets in GC.

*Helicobacter pylori* infection has been reported as a cause of gastric carcinogenesis^28^. *H. Pylori* infection carries cytotoxin-associated antigen A (CagA) into GC cells, after which the CagA protein binds to the Src-homology 2 domain-containing phosphatase 2 (SHP2) domain. SHP2 acts as a positive regulator of Erk activity in GC cells. These findings indicate that the Src protein is necessary for the morphogenetic activity of CagA and oncogenesis of GC cells^28^,^29^. Interestingly, we found that *DDR2*, a member of the Src family, was abundantly overexpressed in the disseminated GC cell lines and in patients. Our results indicated that Src plays consecutive roles in carcinogenesis and in the dissemination of GC cells. Therefore, Src should be regarded as a key molecule in the treatment of GC.

To suppress DDR2 activity in the xenograft mouse model of GC dissemination, we used dasatinib, which is approved for first-line use in patients with chronic myelogenous leukemia and Philadelphia chromosome-positive acute lymphoblastic leukemia^30^. Recently, Yamaguchi *et al.* found that dasatinib suppressed the cooperation between scirrhous GC cells and stromal fibroblasts to form invasive structures, by screening candidate inhibitor molecules^31^. Therefore, our data suggest that DDR2 may be a promising therapeutic target for inhibiting the peritoneal dissemination of GC.

In conclusion, we established a peritoneal dissemination-associated gene-expression signature in GC and revealed that inhibiting DDR2 by dasatinib suppressed peritoneal dissemination. The results of this study provide novel insights into the biology of peritoneal metastasis and suggest a novel targeted therapy that may be potentially used to treat GC.

## Materials and Methods

### Cell lines and cell culture

The human scirrhous GC cell lines HSC-44PE, HSC-39, HSC-58, 44As3, 39As8Luc, 58As1Luc, and 58As9 were described previously^4^,^5^,^11^. These cell lines were cultured in RPMI 1640 supplemented with 10% fetal bovine serum (FBS; Life Technologies, Grand Island, NY), 100 IU/mL penicillin, and 100 mg/mL streptomycin. 58As9 cells were transfected with the pGL4.51 [luc2/CMV/Neo] Vector (E1320 Cloning Systems, Promega, Madison, WI) according to the manufacturer’s instructions. Stable transfectants were selected in Geneticin (400 μg/mL; Invitrogen, Carlsbad, CA), and bioluminescence was used to screen transfected clones for luciferase gene expression using the IVIS system (Xenogen, Alameda, CA). Clones expressing the luciferase gene were designated as 58As9Luc cells.

### Clinical GC samples and DNA and RNA extraction

Primary GC samples were obtained from 200 patients who underwent gastric resection at the Singapore Health Services and deposited in National University Hospital System tissue repositories with signed, informed patient consent. Use of patient samples in this study was approved by the appropriate institutional research ethics review committees. Genomic DNA and RNA were extracted from fresh-frozen tissues using a Qiagen Genomic DNA Extraction Kit (Qiagen, Venlo, Netherlands). Total RNA was extracted using the Trizol reagent (Invitrogen), digested with RNase-free DNase (RQ1 DNase, Promega), and subsequently purified using an RNeasy Mini Kit (Qiagen).

Primary GC samples were also obtained from 195 patients who underwent gastric resection at the Oita Prefectural Hospital (Japan) and the Kyushu University Beppu Hospital (Japan) between 1993 and 2010. All tissue samples were immediately taken from the gastric resections, placed in RNA Later (Takara, Tokyo, Japan), frozen in liquid nitrogen, and stored at −80 °C until RNA extraction. Total RNA was isolated from frozen tissue samples and cell lines using the modified acid-guanidine-phenol chloroform method, or isolated from cultured cell lines using an RNeasy Mini Kit (Qiagen). Moreover, 76 formalin-fixed, paraffin-embedded GC tissues from Kyushu University Beppu Hospital were also used in this study. All patients provided written, informed consent, and the study protocol was approved by the ethics committee of Kyushu University. Experiments with these samples were performed in accordance with the approved guidelines.

### First-strand cDNA synthesis and qRT-PCR experiments

The purity and concentration of all RNA samples were evaluated by measuring the absorbance ratio at 260/280 nm with a NanoDrop ND-1000 spectrophotometer (NanoDrop Technologies, Rockland, DE). Total RNA was reverse-transcribed to cDNA with M-MLV Reverse Transcriptase (Invitrogen). *DDR2* expression was determined using a LightCycler 480 Probes Master Kit (Roche Diagnostics, Basel, Switzerland) according to the manufacturer’s instructions. Detection was achieved using forward and reverse primers with sequences of 5′-acctttggctggactctcct-3′ and 5′-agagcattctgggaatcagg-3′, respectively, and Universal ProbeLibrary Probe #14 (Roche Diagnostics). All qRT-PCRs were run in a LightCycler 480 System II (Roche Diagnostics). The relative mRNA expression levels of *DDR2* were measured using the 2^−ΔΔCT^ method. All qRT-PCR experiments were performed in triplicate.

### Gene-expression and methylation arrays

Cellular RNA-expression levels were profiled using an Agilent DNA microarray. Cyanine (Cy)-labeled cDNA was prepared using T7 linear amplification, after which it was fragmented and hybridized to an oligonucleotide microarray (Whole Human Genome 4 × 44 Agilent G4112F). Fluorescence intensities were obtained using an Agilent DNA microarray scanner and processed by quantile normalization. Methylation analysis with Infinium 450 K arrays was also performed with genomic DNA from cell lines. DNA methylation levels for each CpG site were computed using Genome Studio software as the ratio of the signal intensity for methylated DNA to the total signal intensity (methylated plus unmethylated DNA). The expression and methylation array data obtained with the Singaporean samples was deposited in the Gene Expression Omnibus database under Accession Number GSE30601.

### Designed and synthesized DDR2 targeted shRNA and plasmid

The backbone plasmid pcDNA6.2-GW/EmGFP-miR was from the Block-iT Pol II miR RNAi Expression Vector Kit (Invitrogen). The plasmids pcDNA6.2-GW/EmGFP-miR-DDRshRNA (pCMV-shDDR2) containing shRNA sequences targeting the *DDR2* gene and pcDNA6.2-GW/EmGFP-miR-neg (pCMV-N) containing an unrelated insert were constructed as described in the manual for the Block-iT Pol II miR RNAi Expression Vector Kit. *DDR2* shRNA sequences were designed using online software (http://www.invitrogen.com/rnai). Two double-stranded shRNAs were designed, with the following sequences: sh#1 top: 5′-TGCTGAATGGAGGGTGTGCAAGTCAAGTTTTGGCCACTGACTGACTTGACTTGCACCCTCCATT-3′;sh#1 bottom: 5′-CCTGAATGGAGGGTGCAAGTCAAG TCAGTCAGTGGCCA AAA CTTGACTTGCACACCCTCCATTC-3′;sh#1 top: 5′-TGCTGTCGAGTTGGACCCTTGTTCACGTTTTGGCCACTGACTGACGTGAACAAGTCCAACTCGA-3′; and sh#2 bottom: 5′-CCTGTCGAGTTGGACTTGTTCACGTCAGTCAGTGGCCAAAACGTGAACAAGGGTCCAACTCGAC-3′. The unrelated insert sequence was predicted not to target any known genes.

### *In vitro* cell proliferation, migration, and invasion assays

Cells were seeded at 3,000 cells per well in triplicate 96-wells coated with or without collagen I in 100 μl medium, and cell proliferation was evaluated by performing MTT assays using a Cell Proliferation Kit 1 (Roche Applied Science, Penzberg, Germany) according to the manufacturer’s instructions.

Cell-migration capacity was assessed using the BD Falcon FluoroBlok 24-Multiwell Insert System (BD Bioscience, San Jose, CA). Briefly, cells were seeded in the upper chamber of the 24-well plate in serum-free medium. The lower chamber was filled with 750 μl of medium containing 10% FBS, which acts as a chemoattractant, and incubated in a humidified atmosphere (37 °C and 5% CO_2_). After a 48-hour incubation, the upper chamber was transferred into a second 24-well plate containing 500 μl/well (4 μg/ml) calcein AM in HBSS and incubated for an additional hour (37 °C and 5% CO_2_). Invasive cells that migrated through the membrane were evaluated in a fluorescence plate reader at excitation and emission wavelengths of 485 and 535 nm, respectively. Three independent experiments were performed. Similarly, cell invasion capacities were assessed using the BD BioCoat Tumor Invasion System, 24 Multiwell (BD Bioscience), following the same procedure used for the migration assays.

### Protein detection

Immunohistochemical studies of DDR2 and E-cadherin expression were performed with formalin-fixed, paraffin-embedded surgical sections obtained from patients with GC. For antigen retrieval, tissue sections were deparaffinized and boiled in 0.01 M sodium citrate buffer in a microwave for 10 minutes at 500 W. A rabbit polyclonal antibody against DDR2 (H-108, 1:100 dilution; Santa Cruz Biotechnology, Santa Cruz, CA) and a mouse monoclonal antibody against E-cadherin (36/E-Cadherin, 1:100 dilution; BD Bioscience) were used as primary antibodies. All tissue sections were immunohistochemically stained using the EnVision + Dual Link System-HRP (Dako, Glostrup, Denmark) and were counterstained with hematoxylin. We have determined that the complete H score, which was semi-quantitatively calculated by summing the products of the percentage of cells stained at a given staining intensity (0–100) and the staining intensity score (0, no staining; 1, weak staining; 2, moderate staining; and 3, intense staining). The expression was independently evaluated by 2 of the authors (J. K. and K.M.) in a blinded manner in terms of clinical outcomes and other clinicopathological data.

For immunoblotting, proteins were resolved by sodium dodecyl sulfate-polyacrylamide gel electrophoresis using pre-cast NuPAGE 4%–12% Bis-Tris Gels (Invitrogen), an XCell Sure Lock Mini-Cell Electrophoresis System (Invitrogen), and a Power PAC HC (Bio-Rad Laboratories, Hercules, CA). The resolved proteins were transferred to a nitrocellulose membrane using the iBlot Dry Blotting System (Invitrogen). The membranes were blocked with 5% iBlot (Applied Biosystems, Waltham, MA) and 0.1% Tween-20 (Bio-Rad Laboratories) in phosphate-buffered saline (T-PBS) for 1 hour. The membranes were then incubated with appropriate primary antibodies. Next, the membranes were washed twice for 5 minutes in T-PBS, incubated with an HRP-conjugated secondary antibody for 1 hour, and washed twice for 5 minutes in T-PBS.

Chemiluminescence detection reagents were incubated with the membranes for 1–5 minutes, followed by image acquisition using an Image Quant LAS500 (GE Healthcare, Little Chalfont, UK). Primary antibodies targeting pan actin (Thermo Scientific, Waltham, MA) and DDR2 (H-108, Santa Cruz Biotechnology) were used at a dilution of 1:200. Collagen was stained using the Masson’s Trichrome Kit (Sigma-Aldrich, St. Louis, MO).

### Orthotropic and peritoneal implantation

Six-week-old female BALB/c nu/nu mice were purchased from Kyudo Japan, Inc.; maintained under specific pathogen-free conditions; and provided with sterile food, water, and cages. The ambient light was controlled to provide regular cycles of 12 hours of light and 12 hours of darkness. A total of 1 × 10^6^ cancer cells were transplanted into the gastric wall of each mouse after a laparotomy was performed, as described previously^4^,^5^. and the all animal studies was approved by the ethics committee of Kyushu University and Oita University and dissected all animal procedures were performed in compliance with the Guidelines for the Care and Use of Experimental Animals established by the Committee for Animal Experimentation of Kyushu University and Oita University; these guidelines conform to the ethical standards required by Japanese law and also comply with the guidelines for the use of experimental animals in Japan. To assess the influence of DDR2 on GC dissemination, 1 × 10^6^ cancer cells (58As9Luc control, *DDR2* sh#1, or *DDR2* sh#2 cells) were transplanted into the gastric wall of each mouse. At 28 days post-transplantation, the mice were sacrificed and dissected, and peritoneal dissemination, liver metastasis, and ascites formation were examined. The number of mesentery nodules larger than 5 mm in diameter was also determined. Mice were administered dasatinib (50 mg/kg in 1.0 ml PBS + dimethyl sulfoxide [DMSO]) or PBS + DMSO alone by oral and intraperitoneal injection, thrice a week, beginning at 14 days post-transplantation. *In vivo* photon counting and optical imaging were performed to detect luciferase activity in the mice, using an IVIS system. Cancer cell engraftment into the gastric wall was also evaluated using the IVIS system (Supplementary Fig. 6) at 14 days post-transplantation.

### EEM analysis

To identify genes that mediate peritoneal dissemination, we first compared the expression profiles between matching pairs of parental and derived cell lines (HSC-44PE vs. 44As3; HSC-39 vs. 39As8Luc; HSC-58 vs. 58As1Luc; and HSC-58 vs. 58As9) and between all parental and all derived cell lines (HSC-44PE, HSC-39, HSC-58: 44As3, 39As8Luc, 58As1Luc, and 58As9; Supplementary Fig. 1d). Among these 5 pairs, we identified gene sets that were up- or down-regulated in the derived cell line compared with the parental cell line, using 4 different cutoffs (top 50, 100, 200, and 400 genes). To identify gene sets of clinical relevance among the resulting 40 gene sets, we employed EEM analysis (https://eem.hgc.jp/), which statistically tests whether an input gene set has a coherently expressed subset of significant size in an expression data set of interest. EEM takes two types of inputs: an expression dataset of interest and a gene set library that includes gene sets used as seeds of modules. EEM assumes coherence as an indication of the functionality of gene sets, if a gene set have some function, the member of the gene set should be coherently expressed in the expression dataset. Under this assumption, EEM screens a gene set library for gene sets that have a significantly large coherent subset in the input expression data set. From the gene sets that passed the screening, EEM extracts coherent subsets as expression modules. Furthermore, EEM extracts a significantly coherent subset as an expression module and calculates the mean expression levels of the subset as module activities. By applying EEM to the 40 cell line-derived gene sets on the gastric tumor expression dataset (200 Singapore clinical set), we obtained 18 significant gene sets using a threshold of p < 1.0e−6. Among these gene sets, we selected the most significant genes in terms of overall survival rate and statistical significance tests, based on clinical data from the Singaporean cohort. Overall survival analysis was performed with respect to the overall survival rate and the number of years after the operation. A flow chart of our analysis is presented in Supplementary Fig. 2.

### Statistics

All experiments were repeated at least 3 times. Continuous variables were expressed as the mean ± standard deviation. The results were statistically examined by paired Student’s, Wilcoxon’s, or Fisher’s tests. Overall survival curves were plotted according to the Kaplan–Meier method, and the generalized log-rank test was used to compare the survival curves. The findings were considered statistically significant at a *p* value < 0.05.

## Additional Information

**How to cite this article**: Kurashige, J. *et al.* Integrated Molecular Profiling of Human Gastric Cancer Identifies DDR2 as a Potential Regulator of Peritoneal Dissemination. *Sci. Rep.* **6**, 22371; doi: 10.1038/srep22371 (2016).

## Supplementary Material

Supplementary Information
