# Supplementary material for: Integrated Molecular Profiling of Human Gastric Cancer Identifies DDR2 as a Potential Regulator of Peritoneal Dissemination
Source: Sci Rep. 2016 Mar 3;6:22371. doi: 10.1038/srep22371 (PMC4776110; doi:10.1038/srep22371)
Supplement: Supplementary Information [file srep22371-s1.pdf]

Integrated Molecular Profiling of Human Gastric Cancer Identifies DDR2 as a Potential Regulator of Peritoneal Dissemination

Junji Kurashige[1,2], Takanori Hasegawa[3], Atsushi Niida[4], Keishi Sugimachi[1], Niantao Deng[5], Kosuke Mima[1,2], Ryutaro Uchi[1], Genta Sawada[1,6], Yusuke Takahashi[1,6], Hidetoshi Eguchi[1], Masashi Inomata[7], Seigo Kitano[7], Takeo Fukagawa[8], Mitsuru Sasako[9], Hiroki Sasaki[10], Shin Sasaki[11], Masaki Mori[6], Kazuyoshi Yanagihara[12], Hideo Baba[2], Satoru Miyano[4], Patrick Tan[5], Koshi Mimori[1]

Supplementary Figure Legends

Supplementary Figure 1

Establishment of high peritoneal disseminated gastric cell lines.

(a) Four cell lines with high peritoneal disseminated characteristics were isolated from each parental cell using 12 repeating cycles of orthotropic transplantation directly into the gastric wall in nude mice. (b) Mice implanted with parental HSC-58 cells had few, if any, nodules and no apparent accumulation of ascites (left), and those implanted with disseminated 58As9Luc cells formed disseminated nodules with ascites in the peritoneum (right). Red arrows are tumors on the gastric wall, and the white arrows are peritoneal nodules. (c) H&E staining of histological sections from an orthotopic tumor

formed by HSC-58 at gastric wall (top), an orthotopic tumor formed by 58As9Luc cells at gastric wall (middle), and peritoneal nodules formed by 58As9Luc cells (bottom). Scale bar, 50 µm. (d) 40 sets of the genes are highly (top 50, 100, 200, and 400) upregulated or downregulated between each of the four pairs of parent and derived cell lines and also between all parents and derived.

Supplementary Figure 2

A flow chart of our EEM analysis

At first, we performed EEM analysis of the 40 gene sets in the gastric tumor expression data set (200 Singapore clinical set) and then obtained 18 significant gene sets by a threshold ($p < 1.0e-6$). Second, we additionally applied EEM analysis to 1,981 gene sets that are registered as the GO and curatedCp entries in MSigDB, namely, 2,021 (=40+1,981) gene sets in total. Among these gene sets, only 139 gene sets were selected as significant by a threshold ($p < 1.0e-6$). The significant cell line-derived gene sets were then subject to statistical tests while all significant gene sets were subjected to a clustering analysis.

Supplementary Figure 3

DDR2 had a higher expression in 58As9Luc than in HSC-58 cell line.

(a) The DDR2 expression in HSC-58 and 58As9Luc were confirmed by qRT-PCR, (b) by western blotting, and (c) by immunohistochemistry of the mouse model.

Supplementary Figure 4

Suppression of DDR2 reduced migration and invasion in MKN7 cell line.

(a) The expression of *DDR2* mRNA in 10 gastric cell lines. (b) *DDR2* expression was suppressed in 58As9Luc by two independent shRNA in MKN7. (c) Effects of *DDR2* on migration and (d) invasion in MKN7.

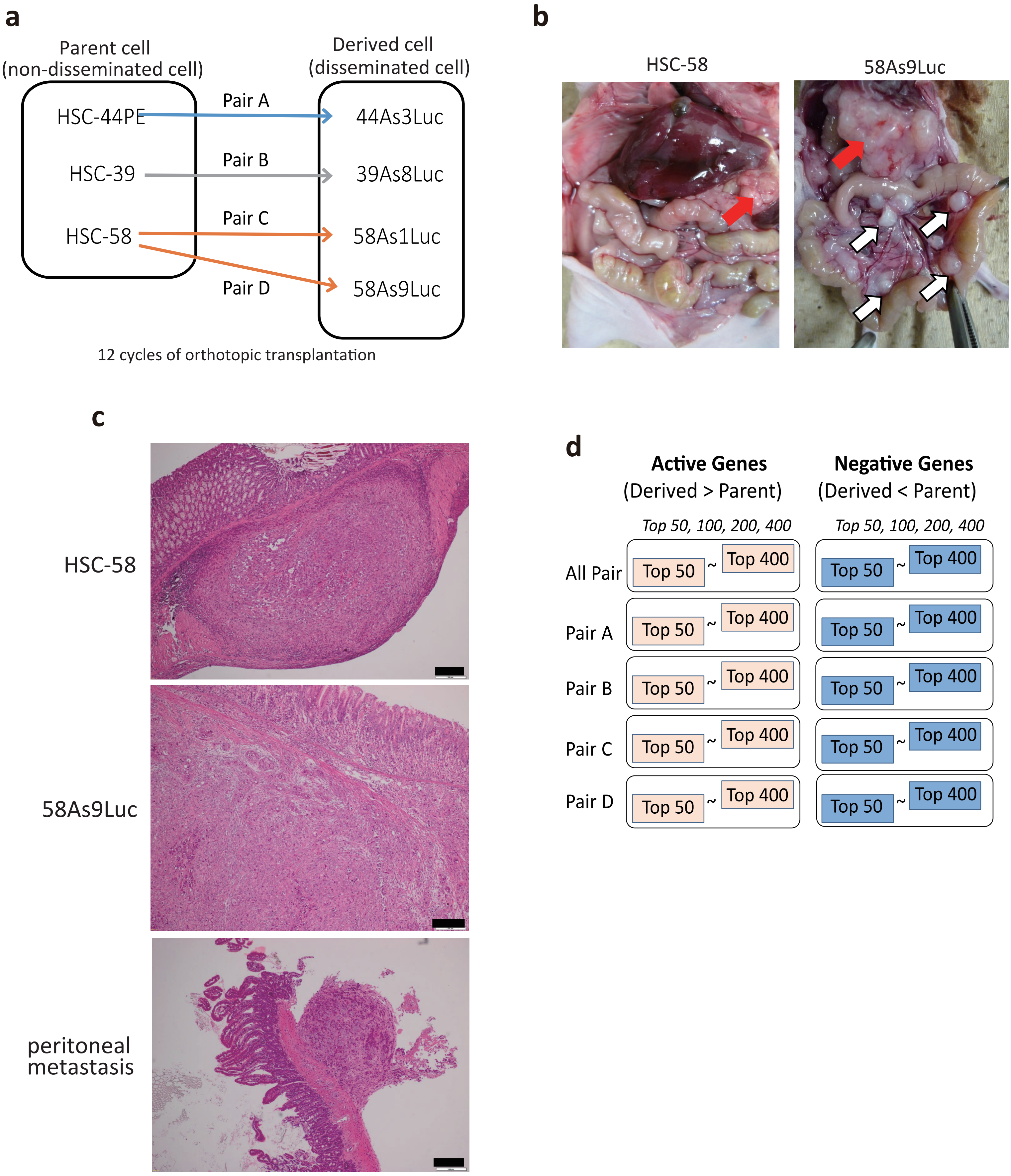
a
Parent cell
(non-disseminated cell)
Derived cell
(disseminated cell)
HSC-44PE
HSC-39
HSC-58
Pair A
Pair B
Pair C
Pair D
44As3Luc
39As8Luc
58As1Luc
58As9Luc
12 cycles of orthotopic transplantation
b
HSC-58
58As9Luc
c
HSC-58
58As9Luc
peritoneal metastasis
d
Active Genes
(Derived > Parent)
Negative Genes
(Derived < Parent)
Top 50, 100, 200, 400
Top 50, 100, 200, 400
All Pair
Pair A
Pair B
Pair C
Pair D
Top 50 ~ Top 400

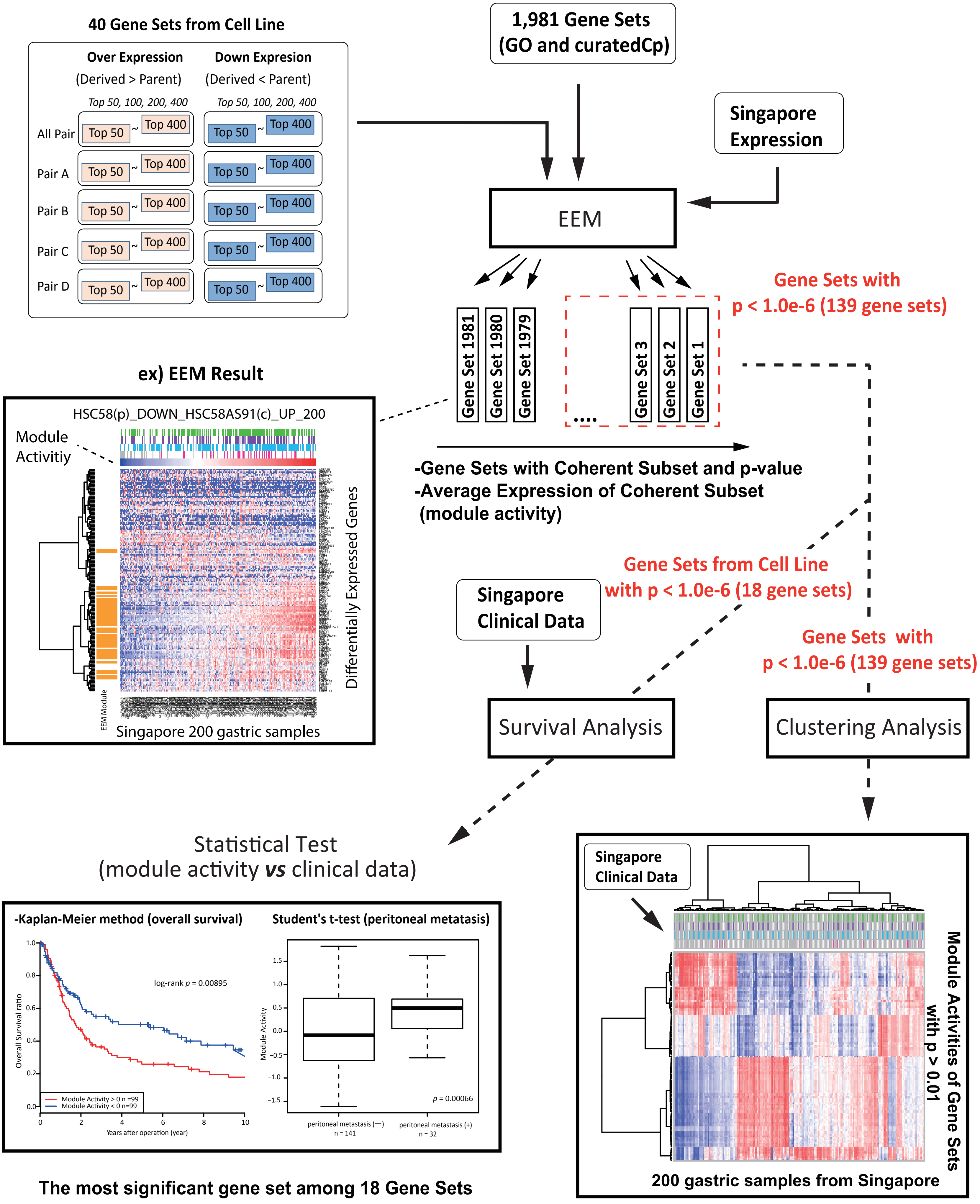
40 Gene Sets from Cell Line
Over Expression
(Derived > Parent)
Top 50, 100, 200, 400
Down Expresion
(Derived < Parent)
Top 50, 100, 200, 400
All Pair
Pair A
Pair B
Pair C
Pair D
Top 50 ~ Top 400
1,981 Gene Sets
(GO and curatedCp)
Singapore Expression
EEM
Gene Set 1981
Gene Set 1980
Gene Set 1979
....
Gene Set 3
Gene Set 2
Gene Set 1
Gene Sets with p < 1.0e-6 (139 gene sets)
ex) EEM Result
HSC58(p)_DOWN_HSC58AS91(c)_UP_200
Module Activitiy
Differentially Expressed Genes
EEM Module
Singapore 200 gastric samples
-Gene Sets with Coherent Subset and p-value
-Average Expression of Coherent Subset
(module activity)
Gene Sets from Cell Line
with p < 1.0e-6 (18 gene sets)
Singapore Clinical Data
Gene Sets with
p < 1.0e-6 (139 gene sets)
Survival Analysis
Clustering Analysis
Statistical Test
(module activity vs clinical data)
-Kaplan-Meier method (overall survival)
log-rank p = 0.00895
Overall Survival ratio
Years after operation (year)
Module Activity > 0 n =99
Module Activity < 0 n=99
Student's t-test (peritoneal metatasis)
Module Activity
p = 0.00066
peritoneal metastasis (—)
n = 141
peritoneal metastasis (+)
n = 32
Singapore Clinical Data
Module Activities of Gene Sets
with p > 0.01
200 gastric samples from Singapore
The most significant gene set among 18 Gene Sets

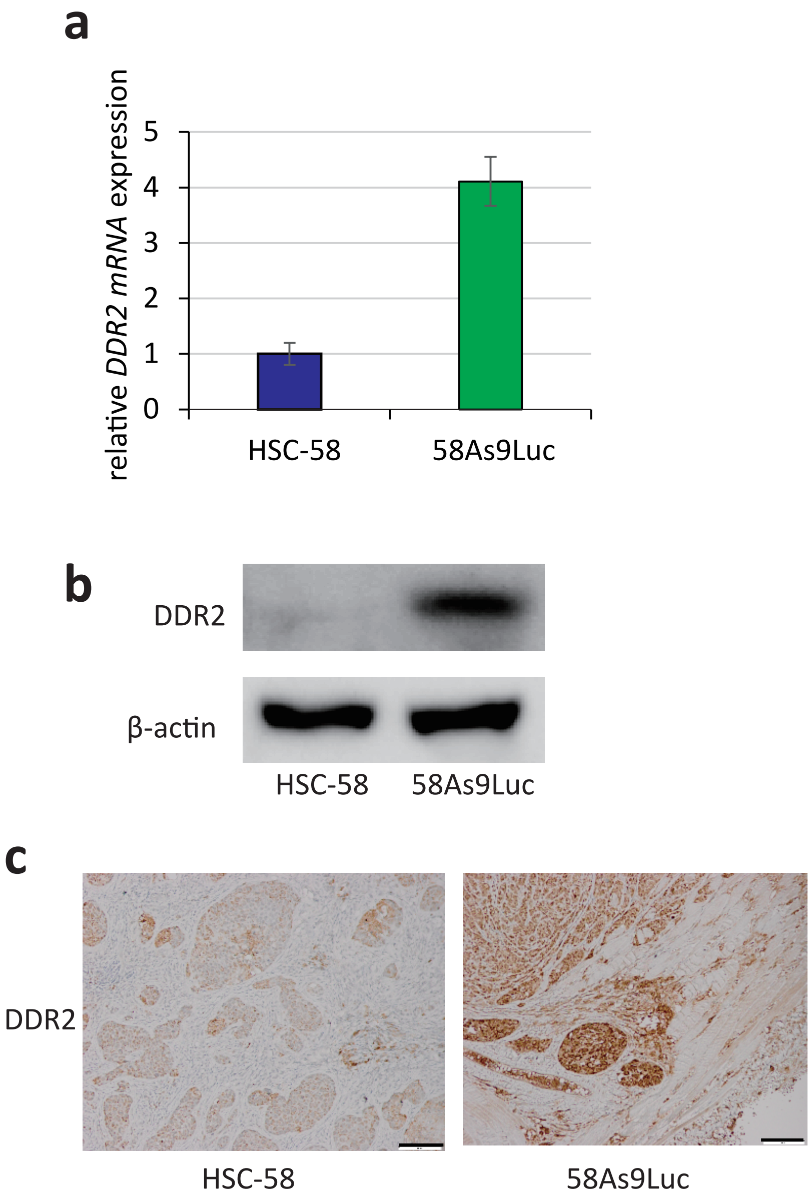
a
relative DDR2 mRNA expression
0
1
2
3
4
5
HSC-58
58As9Luc
b
DDR2
β-actin
HSC-58
58As9Luc
c
DDR2
HSC-58
58As9Luc

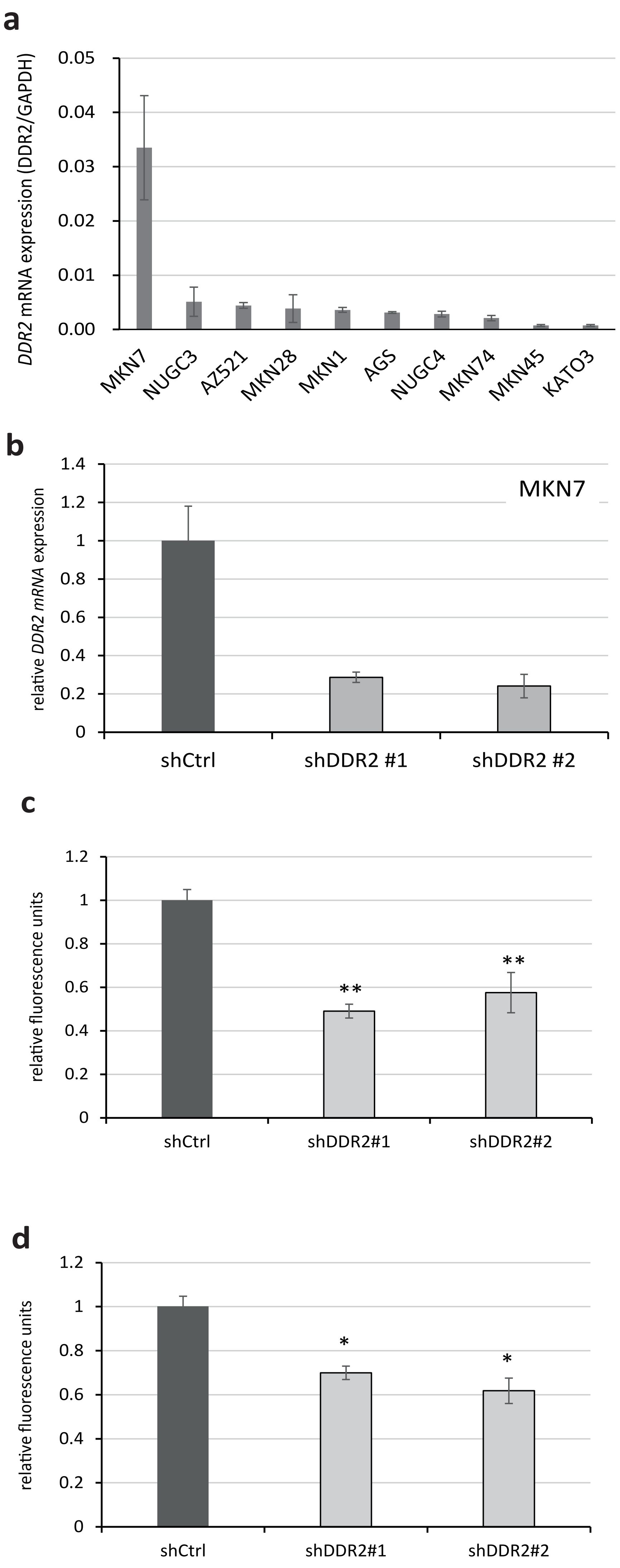
a
DDR2 mRNA expression (DDR2/GAPDH)
0.05
0.04
0.03
0.02
0.01
0.00
MKN7
NUGC3
AZ521
MKN28
MKN1
AGS
NUGC4
MKN74
MKN45
KATO3
b
relative DDR2 mRNA expression
MKN7
1.4
1.2
1
0.8
0.6
0.4
0.2
0
shCtrl
shDDR2 #1
shDDR2 #2
c
relative fluorescence units
1.2
1
0.8
0.6
0.4
0.2
0
**
**
shCtrl
shDDR2#1
shDDR2#2
d
relative fluorescence units
1.2
1
0.8
0.6
0.4
0.2
0
*
*
shCtrl
shDDR2#1
shDDR2#2
